# Obstetric and perinatal outcomes from the follow-up of a multicentre randomized controlled trial investigating time-lapse embryo monitoring

**DOI:** 10.1093/humrep/deaf197

**Published:** 2025-10-26

**Authors:** D C Kieslinger, C G Vergouw, F von Estorff, L Ramos, B Arends, M H J M Curfs, E Slappendel, E H Kostelijk, M H E C Pieters, D Consten, M O Verhoeven, D E Besselink, F Broekmans, B J Cohlen, J M J Smeenk, S Mastenbroek, C H de Koning, Y M van Kasteren, E Moll, J van Disseldorp, E A Brinkhuis, E A M Kuijper, W M van Baal, H G I van Weering, P J Q van der Linden, M H Gerards, P M Bossuyt, M van Wely, C B Lambalk

**Affiliations:** IVF Center, Amsterdam UMC—VUmc, Amsterdam, The Netherlands; IVF Center, Amsterdam UMC—VUmc, Amsterdam, The Netherlands; IVF Center, Amsterdam UMC—VUmc, Amsterdam, The Netherlands; IVF Center, Radboudumc, Nijmegen, The Netherlands; IVF Center, UMC Utrecht, Utrecht, The Netherlands; Isala Fertility Center, Isala, Zwolle, The Netherlands; Center for Fertility, Nij Geertgen, Elsendorp, The Netherlands; IVF Center, Amsterdam UMC—VUmc, Amsterdam, The Netherlands; IVF Center, UMC Utrecht, Utrecht, The Netherlands; IVF Center, ETZ, Tilburg, The Netherlands; IVF Center, Amsterdam UMC—VUmc, Amsterdam, The Netherlands; IVF Center, Radboudumc, Nijmegen, The Netherlands; IVF Center, UMC Utrecht, Utrecht, The Netherlands; Isala Fertility Center, Isala, Zwolle, The Netherlands; IVF Center, ETZ, Tilburg, The Netherlands; Center for Reproductive Medicine, Amsterdam UMC—AMC, Amsterdam, The Netherlands; Center for Fertility, Tergooi MC, Blaricum, The Netherlands; Center for Fertility, NWZ, Alkmaar, The Netherlands; Center for Fertility, OLVG, Amsterdam, The Netherlands; Center for Fertility, St. Antonius, Nieuwegein, The Netherlands; Center for Fertility, Meander MC, Amersfoort, The Netherlands; Center for Fertility, Spaarne Gasthuis, Haarlem, The Netherlands; Center for Fertility, Flevo Hospital, Almere, The Netherlands; Center for Fertility, RKZ, Beverwijk, The Netherlands; Department of Obstetrics, Gynaecology and Reproductive Medicine, Deventer Ziekenhuis, Deventer, The Netherlands; Center for Fertility, Diakonessenhuis, Utrecht, The Netherlands; Epidemiology & Data Science, Amsterdam UMC, Amsterdam, The Netherlands; Center for Reproductive Medicine, Amsterdam UMC—AMC, Amsterdam, The Netherlands; IVF Center, Amsterdam UMC—VUmc, Amsterdam, The Netherlands

**Keywords:** time-lapse monitoring, safety, embryo culture, birth weight, obstetric and perinatal outcomes, IVF, machine learning

## Abstract

**STUDY QUESTION:**

Does uninterrupted culture in a time-lapse incubator with or without a commercially available machine learning embryo selection algorithm result in comparable obstetric and perinatal outcomes as interrupted culture and morphological embryo selection?

**SUMMARY ANSWER:**

The application of uninterrupted culture in a time-lapse incubator with and without the use of an embryo selection algorithm is comparable to interrupted embryo culture and morphological embryo selection in terms of obstetric and perinatal results.

**WHAT IS KNOWN ALREADY:**

There is very limited evidence regarding the safety of time-lapse monitoring (TLM) from prospective randomized controlled trials (RCT). Recent RCTs have demonstrated that the application of TLM does not increase (cumulative) live birth rates or shorten the time to pregnancy within 1 year. Although most studies only report pregnancy rates, the safety of this commonly used method is also relevant for decision-making.

**STUDY DESIGN, SIZE, DURATION:**

The obstetric and perinatal outcomes of patients scheduled for Day 3 single embryo transfer who participated in a multicentre RCT on TLM were studied (SelecTIMO trial). Three groups were compared: (i) TLE: embryo selection based on a commercially available Day 3 TLM algorithm, used adjunctively with morphology, and uninterrupted culture. (ii) TLR: routine morphological embryo selection and uninterrupted culture. (iii) CON: routine morphological embryo selection and interrupted culture.

**PARTICIPANTS/MATERIALS, SETTING, METHODS:**

In total, 1731 IVF/ICSI patients undergoing their first, second, or third oocyte retrieval cycle were randomized. Obstetric and perinatal data were registered for all pregnancies occurring after fresh and frozen embryo transfers associated with the initial oocyte retrieval cycle as well as natural conceptions within 1 year. Serious pregnancy complications and birth weight were considered main safety outcomes. Mean differences (MD) and age-adjusted relative risks (RR_adj_) and mean differences with 95% CI were calculated for TLE and TLR versus CON.

**MAIN RESULTS AND THE ROLE OF CHANCE:**

A total of 827 women gave birth to a singleton during the follow-up period (TLE = 275, TLR = 278, CON = 274; *P* = 0.99). Of the 827 women who gave birth to a singleton, 497 deliveries originated from a fresh embryo transfer (60%), 294 from a frozen embryo transfer (36%), and 36 women conceived naturally (4%), with similar proportions in each study group. The proportion of women with serious pregnancy complications was comparable across the three groups (TLE vs CON: RR_adj_ 0.95, 95% CI 0.65–1.40 and TLR vs CON: RR_adj_ 1.03, 95% CI 0.70–1.50; *P* = 0.89). Mean (SD) gestational age at birth was 39.4 (1.9) weeks, 39.5 (1.5) weeks, and 39.3 (1.9) weeks, respectively. We found no evidence of differences in preterm and very preterm births between groups. Mean (SD) weight at birth was 3413 (588) g, 3412 (588) g, and 3377 (578) g, respectively (TLE vs CON: MD 34, 95% CI −62 to 129 and TLR vs CON: MD 32, 95% CI −635 to 120; *P* = 0.70). We did not observe substantial differences in babies with low and very low birth weight. Health problems immediately after delivery were reported for eight babies in the TLE group, 12 in the TLR group, and 11 in the CON group. Major congenital malformations occurred in four children in the TLE group, four in the TLR group, and seven in the CON group. Minor congenital malformations occurred in five children in the TLE group, three in the TLR group, and five in the CON group.

**LIMITATIONS, REASONS FOR CAUTION:**

This study reports safety outcomes for one type of time-lapse incubator, however, more systems are currently available.

**WIDER IMPLICATIONS OF THE FINDINGS:**

Our results suggest that uninterrupted time-lapse culture with and without embryo selection based on machine learning can be regarded as safe compared to interrupted embryo culture and routine morphological selection in terms of obstetric and perinatal risks.

**STUDY FUNDING/COMPETING INTEREST(S):**

The authors received a grant from the Netherlands Organisation for Health Research and Development (ZonMw) for the execution of the SelecTIMO study (Health Care Efficiency Research programme grant 843001602). Merck (Germany and The Netherlands) supplied the six time-lapse incubators, funded the laboratory adjustments, and provided technical support and training to laboratory personnel before and during the study. D.C.K. received the Fertility Society of Australia exchange award. The following declarations of interest were made outside of the submitted work: F.B. reports additional financial support for the LUMO trial from Besins Healthcare Monaco, fellowship grants for ongoing basic research from Merck, consulting fees and payment or honoraria from Merck, Besins, and Ferring, and is member of the DSMB of the POISE study UK. J.M.J.S. has received grants or contracts from Ferring BV and Merck (payments to ETZ in both cases); consulting fees for an advisory board from Ferring BV; speakers fee from Merck BV; and support for conference attendance from Ferring BV, Merck, and Goodlife. M.v.W. is Senior Editor of Cochrane and Editor-in-Chief of Human Reproduction Update. C.B.L. reports a speakers honorarium from Organon (The Netherlands) and was Editor In Chief for Human Reproduction at the time of submitting this manuscript.

**TRIAL REGISTRATION NUMBER:**

NTR5423: ICTRP Search Portal (who.int).

## Introduction

IVF laboratories try to improve clinical success rates by offering the latest innovations and techniques to their patients. It is of utmost importance to provide the best possible culture environment for the developing embryos and to select the highest quality embryo for transfer to achieve a healthy pregnancy and live birth. Time-lapse monitoring (TLM) is a technique that has been introduced to IVF centres worldwide a decade ago, even though there was insufficient evidence for its effectiveness and safety available at the time. Two reasons were advocated why TLM would optimize IVF procedures and improve clinical results. First, built-in cameras obviate the need to remove embryos from the incubator for morphological assessments, thereby providing very stable culture conditions (Aparicio *et al.*, 2013). Second, TLM generates detailed information on embryo development, which can be used by embryologists, artificial intelligence, or computer software for optimal embryo selection (Wong *et al.*, 2013; Berntsen *et al.*, 2022).

Recent randomized controlled trials (RCT) have demonstrated that the application of TLM does not increase (cumulative) live birth rates or decrease the time to pregnancy within 1 year (Goodman *et al.*, 2016; Ahlstrom *et al.*, 2022; Kieslinger *et al.*, 2023; Bhide *et al.*, 2024; Illingworth *et al.*, 2024). However, most data on the safety of TLM stem from retrospective studies or small RCTs and there is limited evidence from large RCTs.

A retrospective follow-up study of an RCT concluded that there were no differences between time-lapse and conventional incubators regarding the obstetric and perinatal outcomes of singleton ongoing pregnancies (Insua *et al.*, 2017). Another RCT also reported no differences regarding perinatal outcomes (Kovacs *et al.*, 2019). Both studies compared different types of conventional incubators to a time-lapse incubator. A pragmatic multicentre RCT on TLM reported reassuring results regarding major congenital anomalies and adverse events (Bhide *et al.*, 2024).

Recently, 1731 IVF/ICSI patients participated in a three-armed, multicentre, double-blind, RCT (Kieslinger *et al.*, 2023). All embryos were cultured in the same time-lapse incubator (Geri+). The aim of the current study was to compare the safety of uninterrupted embryo culture using TLM (with and without the use of machine learning for embryo selection) and interrupted culture (with morphological embryo selection). To assess the safety, we evaluated the obstetric and perinatal data for all singleton ongoing pregnancies occurring within 1 year after fresh single embryo transfer (SET) and single frozen embryo transfer (FET) associated with the initial oocyte retrieval cycle, as well as natural conceptions.

## Materials and methods

### Study setting, design, and population

Three groups were compared: (i) TLE (Time-Lapse Eeva^®^): embryo selection based on the Eeva^®^ Test (a Day 3 TLM algorithm based on machine learning, used adjunctively with morphology to predict blastocyst formation without the need for extended culture until Day 5) and uninterrupted culture; (ii) TLR (Time-Lapse Routine): routine morphological embryo selection and uninterrupted culture; and (iii) CON (Control group): routine morphological embryo selection and interrupted culture.

The embryos in all three groups were cultured in a Geri+ time-lapse incubator (Genea Biomedx, Sydney, NSW, Australia) under 5% O_2_ and 5–6% CO_2_.

Patients were recruited at five independent IVF laboratories and 10 associated fertility clinics in the Netherlands between June 2017 and March 2020 (Kieslinger *et al.*, 2023). The trial protocol was first approved on 22 December 2016 by the Central Committee on Research involving Human Subjects (The Hague, The Netherlands) and by the board of directors of each participating clinic. Participating couples provided written informed consent. The study was registered at Trial Search on 8 September 2015 (NTR5423: ICTRP Search Portal (who.int)).

Couples in their first, second, or third IVF or ICSI oocyte retrieval cycle were invited to participate if they were planning to have a fresh SET. Couples could only participate once. Exclusion criteria were: planned double embryo transfer, planned freeze all cycle without a fresh embryo transfer, participation in another scientific study, use of donor gametes, preimplantation genetic testing and the use of thawed oocytes. All women were 42 years or younger, since IVF treatment is covered by Dutch health insurances until this age. Randomization was conducted centrally in Castor (a web-based, online randomization tool) and stratified by laboratory site and oocyte retrieval cycle number.

In the TLE and TLR groups, zygotes were transferred to Geri+ culture dishes after fertilization check on Day 1 and kept in the incubator without disturbances until Day 3, thereby providing uninterrupted culture conditions. In the CON group, the standard dishes of each laboratory were used for embryo culture, which were removed from the incubator two additional times for embryo assessments under a conventional microscope between Day 1 and Day 3, thereby providing interrupted culture conditions. Embryos were cultured in individual medium droplets in the CON group (20–50 µl) and in group culture microwell droplets (80 µl) in the TLE and TLR groups.

Geri+ incubator software version 5.3-6.0, Geri connect software version 1.0-2.0 and Eeva^®^ system version 3.0-3.1 were used in the trial. Images in 11 focal planes were captured for each embryo in the TLE and TLR groups every 5 min with a total light exposure time of ∼125 s per day per embryo. Fresh embryo transfers were conducted on Day 3. Embryo selection in the TLE group was based on the results of the EEVA^®^ Test in combination with morphology. Embryo selection in the TLR and CON groups was based on routine morphology. All embryos were cultured in the Geri+ incubator until the moment of cryopreservation. Embryos with the highest Eeva^®^ Test result (TLE) or best morphology (TLR and CON) were frozen and thawed first. All laboratories adhered to the study protocol, but other than that, used their own laboratory protocols, culture media, and cryopreservation techniques.

Additional details concerning randomization and IVF protocols can be found in our previously published article (Kieslinger *et al.*, 2023).

### Data sources

Obstetric and perinatal data were primarily based on medical records and entered by research personnel and fertility doctors of each participating clinic in Castor, a web-based, clinical data management platform. The outcomes of all pregnancies occurring after fresh embryo transfers and FETs associated with the initial oocyte retrieval cycle as well as natural conceptions within 1 year were recorded. Additionally, patients were asked to fill in an online questionnaire about their pregnancy, delivery, and child (Castor). Apgar score results were based on data from questionnaires only. Data entered by research personnel and information reported by patients were cross-checked, harmonized, and subsequently categorized.

### Outcome measurements

The following obstetric outcomes (where applicable with or without hospital admission) were recorded separately: premature contractions (before 37 weeks of gestation), premature rupture of membranes (before 37 weeks of gestation), premature placental abruption, placenta praevia, hypertension, pre-eclampsia, gestational diabetes, postpartum bleeding (>1.5 l), and other serious pregnancy-related health problems (hyperemesis gravidarum, pelvic floor disorders, and others). The occurrence of at least one of the above complications including preterm birth before 37 weeks was registered as serious pregnancy complications overall. The following modes of delivery were recorded: spontaneous start of delivery, induced delivery, ventouse delivery, and caesarean section.

Perinatal outcomes were live birth, stillbirth/intrauterine death, term birth after ≥37 weeks, preterm birth (<37 weeks), and very preterm birth (<32 weeks), weight at birth, low birth weight (<2500 g), very low birth weight (<1500 g), gestational age at birth, Apgar score at 1 and 5 min, minor and major congenital malformations, and health problems directly after birth, such as dysmaturity, breathing problems, or neonatal hypoglycaemia. Congenital malformations were defined according to the manual from the World Health Organization published in 2020: Major congenital malformations are structural changes that have significant medical, surgical, social, or cosmetic consequences for the affected individual, and typically require medical intervention. Examples include spina bifida, anencephaly, heart defects, and orofacial clefts. Minor congenital malformations are structural changes that pose little or no significant health problem and tend to have limited social or cosmetic consequences for the affected individual. Examples of minor anomalies include single palmar crease and clinodactyly (mild curvature of a finger) (World Health Organization, 2020).

### Sample size considerations

The clinical outcomes of 1731 randomized couples were included in this analysis. The sample size for this study was not aimed at safety outcomes but based on the ability to demonstrate an absolute difference of 7.5% or more in cumulative ongoing pregnancy (Kieslinger *et al.*, 2023). For obstetric outcomes, we considered serious pregnancy complications overall to be our main outcome. As we had at least 274 singleton deliveries in each allocated group, this sample size would allow us enough power to detect a difference of 10% compared to a control proportion of 20%. For perinatal outcomes, we considered the weight of the neonate our main outcome; with at least 274 singletons per group, this would allow us to detect a mean difference of 144 g or more (expected weight of 3400 and SD of 600).

### Statistical analysis

In view of the many outcomes and comparisons, we present data descriptively and calculated age-adjusted effect measures with 95% confidence intervals for TLE and TLR compared to CON for singleton pregnancies only. *P*-values were calculated only for the main safety outcomes. For linear outcomes, we used a generalized linear model adjusted for maternal age to estimate mean differences between TLE and CON and TLR and CON. In case the outcome had a skewed distribution, a gamma with log link model was used. For binary outcomes, a generalized linear model using a negative binomial with log link adjusted for maternal age was applied to estimate adjusted relative risks between TLE and CON and TLR and CON.

Regression analyses of weight percentiles of children born after fresh embryo transfers and FETs were conducted. *Z*-scores for birth weight by gestational age in weeks were placed in the Dutch reference curves for boys and girls. Percentiles for birth weight by gestational age were analysed for fresh embryo transfers and FETs and gender separately. All outcomes were reported for singleton pregnancies.

## Results

Our analysis is based on 873 women who had an ongoing pregnancy during the follow-up period of 1 year. In total, 39 women experienced a late miscarriage, induced abortion, stillbirth, or intrauterine death, or were lost to follow-up after an ongoing pregnancy was confirmed. Of these 39 women, 6 had a live birth after a subsequent treatment cycle within 1 year, bringing the total to 840 women who delivered during the 1-year follow-up period. There were 827 singleton and 13 twin deliveries. One neonatal death occurred following the delivery of a singleton in the CON group.

Baseline characteristics of all patients who had a singleton live birth are presented in Table 1.

**Table 1.** Baseline characteristics of all women who had a singleton live birth.

	**TLE group n = 275**	TLR group n = 278	CON group n = 274
Female age (years)	33.5 (4.0)	33.0 (3.8)	33.6 (3.9)
Female BMI (kg/m^2^)	24.2 (4.8)	24.0 (4.0)	24.7 (4.7)
Smoking behaviour (woman)			
Yes	27 (9.6%)	30 (10.6%)	31 (11.2%)
No	241 (85.8%)	240 (85.1%)	236 (85.2%)
Unknown	13 (4.6%)	12 (4.3%)	10 (3.6%)
Smoking behaviour (male)			
Yes	51 (18.1%)	51 (18.1%)	48 (17.3%)
No	201 (71.5%)	201 (71.3%)	207 (74.7%)
Unknown	29 (10.3%)	30 (10.6%)	22 (7.9%)
Pregnancy history			
Previous ongoing pregnancy	89 (31.7%)	73 (25.9%)	74 (26.7%)
Previous miscarriage abortion, EUG	84 (29.9%)	71 (25.2%)	79 (28.5%)
Reason for IVF/ICSI			
Male factor	115 (40.9%)	122 (43.3%)	115 (41.5%)
Female factor	70 (24.9%)	70 (24.8%)	64 (23.1%)
Male and female factor	15 (5.3%)	16 (5.7%)	15 (5.4%)
Unexplained	74 (26.3%)	70 (24.8%)	77 (27.8%)
Other	7 (2.5%)	4 (1.4%)	6 (2.2%)
Duration of infertility (months)	35.6 (23.4)	29.4 (18.9)	33.1 (28.6)
Total FSH dose	1616.8 (2639.0)	1500.6 (3363.4)	1597.3 (965.2)
Pre-treatment			
Yes (OC or progestative)	221 (78.6%)	220 (78.0%)	221 (79.8%)
No	60 (21.4%)	62 (22.0%)	56 (20.2%)
Premature LH surge prevention			
GnRH antagonist	52 (18.6%)	49 (17.4%)	46 (16.6%)
GnRH agonist	228 (81.4%)	233 (82.6%)	231 (83.4%)
Stimulation protocol			
Gonal-F	255 (90.7%)	242 (85.8%)	244 (88.1%)
Menopur	7 (2.5%)	15 (5.3%)	6 (2.2%)
Puregon	1 (0.4%)	0 (0.0%)	1 (0.4%)
Fostimon	5 (1.8%)	7 (2.5%)	6 (2.2%)
Other	13 (4.6%)	18 (6.4%)	20 (7.2%)
Fertilization method			
IVF	158 (56.2%)	140 (49.8%)	149 (53.8%)
ICSI	118 (42.0%)	134 (47.7%)	126 (45.5%)
Both	5 (1.8%)	7 (2.5%)	2 (0.7%)
Oocyte retrieval number			
Oocyte retrieval 1	264 (94.0%)	266 (94.3%)	251 (90.6%)
Oocyte retrieval 2	13 (4.6%)	13 (4.6%)	21 (7.6%)
Oocyte retrieval 3	4 (1.4%)	3 (1.1%)	5 (1.8%)

Continuous variables are presented as mean (SD); Categorical variables are presented as sample size (proportion).

TLE, embryo selection based on a commercially available Day 3 TLM algorithm, used adjunctively with morphology, and uninterrupted culture; TLR, routine morphological embryo selection, and uninterrupted culture; CON, routine morphological embryo selection and interrupted culture; OC, oral contraceptive.

### Obstetric outcomes

Table 2 reports the cumulative outcomes for all singleton live births. No statistically significant differences between the three groups were observed for the percentage of serious pregnancy complications overall (TLE = 20.0% (55/275), TLR = 21.6% (60/278), CON = 21.2% (58/274); *P* = 0.89). Comparing TLE and TLR with CON, the age-adjusted RR did not indicate significant differences in premature contractions, premature rupture of membranes, premature placental abruption, placenta praevia, high blood pressure, pre-eclampsia, gestational diabetes, postpartum bleeding, or other pregnancy-related health problems.

**Table 2.** Cumulative results for all singletons.

	TLE group (n = 571)	TLR group (n = 575)	CON group (n = 572)	Overall *P*-value	TLE vs CON	TLR vs CON
					**Adjusted RR** **	**Adjusted RR** **
Live birth	275/571 (48.2%)	278/575 (48.3%)	274/572 (47.9%)	0.99	1.01 (0.82–1.24)	1.00 (0.81–1.23)
Stillbirth/intrauterine death	3/571 (0.5%)	3/575 (0.5%)	2/572 (0.3%)		1.47 (0.24–8.81)	1.48 (0.24–8.88)
Late miscarriage	6/571 (1.1%)	4/575 (0.7%)	1/572 (0.2%)		6.27 (0.75–52.30)	4.13 (0.46–37.16)
Induced abortion	3/571 (0.5%)	9/575 (1.6%)	7/572 (1.2%)		0.45 (0.12–1.76)	1.33 (0.49–3.61)
**Obstetric outcomes**						
Serious pregnancy complications overall	55/275 (20.0%)	60/278 (21.6%)	58/274 (21.2%)	0.89	0.95 (0.65–1.40)	1.03 (0.70–1.50)
Premature contractions (before 37 weeks pregnancy)	2/275 (0.7%)	3/278 (1.1%)	2/274 (0.7%)		0.97 (0.14–6.97)	1.39 (0.23–8.40)
Premature rupture of membranes (before 37 weeks pregnancy)	6/275 (2.2%)	4/278 (1.4%)	4/274 (1.5%)		1.49 (0.415.35)	0.98 (0.23–3.92)
Premature placental abruption	1/275 (0.4%)	0/278 (0.0%)	0/274 (0.0%)		NA	NA
Placenta praevia	1/275 (0.4%)	6/278 (2.2%)	4/274 (1.5%)		0.25 (0.03–2.24)	1.46 (0.41–5.25.)
High blood pressure (Hypertension)	19/275 (6.9%)	24/278 (8.6%)	17/274 (6.2%)		1.11 (0.57–2.18)	1.38 (0.73–2.63)
Pre-eclampsia	6/275 (2.2%)	16/278 (5.8%)	7/274 (2.6%)		0.85 (0.25–2.54)	2.19 (0.87–5.38)
Gestational diabetes	11/275 (4.0%)	10/278 (3.6%)	11/274 (4.0%)		0.99 (0.42–2.33)	0.89 (0.37–2.12)
Other pregnancy-related health problems***	14/275 (5.1%)	15/278 (5.4%)	18/274 (6.6%)		0.83 (0.42–1.64)	0.88 (0.45–1.72)
Postpartum bleeding (>1.5 l)	3/275 (1.1%)	3/278 (1.1%)	5/274 (1.8%)		0.59 (0.14–2.48)	0.59 (0.14–2.46)
Spontaneous start of delivery	132/275 (48.0%)	120/278 (43.2%)	124/274 (45.3%)		1.06 (0.79–1.43)	0.96 (0.71–1.29)
Induced delivery	48/275 (17.5%)	76/278 (27.3%)	61/274 (22.3%)		0.78 (0.52–1.19)	1.23 (0.84–1.79)
Ventouse delivery	21/275 (7.6%)	29/278 (10.4%)	26/274 (9.5%)		0.79 (0.44–1.46)	1.08 (0.62–1.88)
Caesarean section	39/275 (14.2%)	51/278 (18.3%)	51/274 (18.6%)		0.76 (0.39–1.49)	1.06 (0.57–1.98)
**Perinatal outcomes**						
Term birth (≥37 weeks)	255/275 (92.7%)	261/278 (93.9%)	257/274 (93.5%)		0.98 (0.78–1.22)	1.01 (0.80–1.2820)
Preterm birth (<37 weeks) (n = 54)	20/275 (7.3%)	17/278 (6.1%)	17/274 (6.2%)		1.18 (0.62–2.21)	0.99 (0.51–1.89)
Fresh ET (n = 497)	12/162 (7.4%)	10/161 (6.2%)	8/174 (4.6%)			
FET (n = 294)	6/97 (6.2%)	7/108 (6.5%)	7/89 (7.9%)			
Natural conception (n = 36)	2/16 (12.5%)	0/9 (0.0%)	2/11 (18.2%)			
Very preterm birth (<32 weeks)	4/275 (1.5%)	1/278 (0.4%)	2/274 (0.7%)		1.95 (0.35–10.76)	0.7 (0.04–5.19)
Weight at birth in g as mean (SD) (n = 816)*	3413 (588)	3412 (588)	3377 (578)	0.70	MD 34 (−62 to 129)	MD 32 (−635 to 128)
Low birth weight (<2500 g)	17/270 (6.3%)	12/275 (4.4%)	16/271 (5.5%)		1.08 (0.53–2.18)	0.764 (0.35–1.638)
Very low birth weight (<1500 g)	2/270 (0.7%)	2/275 (0.7%)	3/271 (1.1%)		0.67 (0.11–4.05)	0.66 (0.10–3.99)
Gestational age at birth as mean (SD) (n = 815)*	39.4 (1.9)	39.5 (1.5)	39.3 (1.9)		#MD 0.09 (−0.211 to 0.40)	#MD 0.17 (−0.14 to 0.47)
Fresh ET (n = 494)	39.4 (1.9)	39.4 (1.5)	39.4 (1.7)			
FET (n = 285)	39.5 (2.0)	39.7 (1.6)	39.3 (2.2)			
Natural conception (n = 36)	39.5 (1.9)	39.3 (0.8)	39.0 (2.2)			
Apgar score at 1 min as mean (SD) (n = 469)	8.67 (1.66)	8.53 (1.79)	8.20 (2.35)		MD 0.47 (0.03–0.91)	MD 0.34 (−0.08 to 0.77)
Apgar score at 5 min (n = 456)	9.59 (1.35)	9.58 (1.23)	9.44 (1.34)		MD 0.37 (−0.01 to 0.75)	MD 0.36 (−0.00 to 0.73)
Apgar score <4 at 5 min (n = 456)	1/137 (0.7%)	3/160 (1.9%)	2/159 (1.3%)			
Major congenital malformations	4/275 (1.5%)	4/278 (1.4%)	7/274 (2.5%)		0.57 (0.17–1.99)	0.44 (0.11–1.72)
Minor congenital malformations	5/275 (1.8%)	3/278 (1.1%)	5/274 (1.8%)		0.99 (0.28–3.47)	0.58 (0.14–2.48)
Health problems directly after birth	8/275 (2.9%)	12/278 (4.3%)	11/274 (4.0%)		0.76 (0.31–1.88)	1.04 (0.44–2.45)

Data are n/N (%) unless otherwise specified. Live birth was defined as a delivery resulting in a liveborn child. Late miscarriage (>12 and <21 weeks) was defined as pregnancy loss after ongoing pregnancy was determined. Each woman could have multiple pregnancies and miscarriages due to the follow-up period of 1 year. Six women had a live birth after a late miscarriage or induced abortion.

* Gestational age and weight at birth are not known for all live births.

** All effect estimates were adjusted for age. #gamma link was used as gestational age has a skewed distribution.

*** Other pregnancy-related health problems included hyperemesis gravidarum, pelvic floor disorders, overstimulation, and others.

TLE, embryo selection based on a commercially available Day 3 TLM algorithm, used adjunctively with morphology, and uninterrupted culture; TLR, routine morphological embryo selection, and uninterrupted culture; CON, routine morphological embryo selection and interrupted culture; ET, embryo transfer; FET, frozen ET; RR, relative risk; MD, mean difference.

Regarding the delivery mode, the number of women who had a spontaneous start of delivery, induced delivery, ventouse delivery, or caesarean section was comparable between the groups.

### Perinatal outcomes

Of the 827 women who gave birth to a singleton, 497 deliveries originated from a fresh embryo transfer (60%), 294 from a FET (36%), and 36 women conceived naturally (4%), with similar proportions in each study group. The 12-month cumulative live birth rate was 48.2% (275/571) in the TLE group, 48.3% (278/575) in the TLR group, and 47.9% (274/572) in the CON group (*P* = 0.99). Stillbirth/intrauterine death rates were comparable between the three groups (TLE = 0.5% (3/571), TLR = 0.5% (3/575), CON = 0.3% (2/572)).

The preterm birth rate (<37 weeks) was 7.3% (20/275) in the TLE group, 6.1% (17/278) in the TLR group, and 6.2% (17/274) in the CON group. The very preterm birth rate (<32 weeks) was 1.5% (4/275) in the TLE group, 0.4% (1/278) in the TLR group, and 0.7% (2/274) in the CON group.

**Figure 1.**
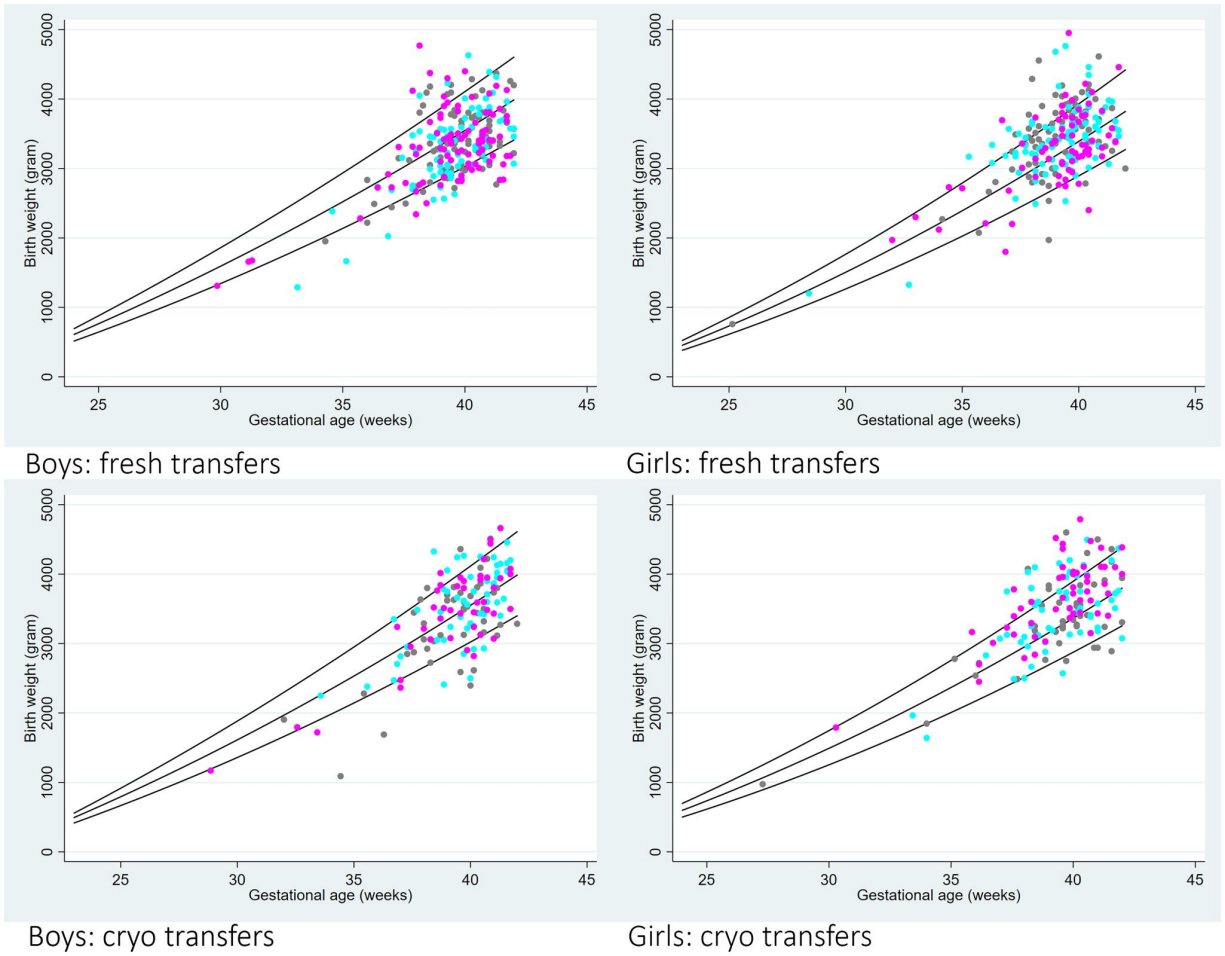
**Birth weights plotted against gestational age in weeks placed in the Dutch reference curves for boys and girls.** Coloured dots represent birthweight in the TLE group (pink), TLR group (turquoise), and CON group (grey). TLE, embryo selection based on a commercially available Day 3 TLM algorithm, used adjunctively with morphology, and uninterrupted culture; TLR, routine morphological embryo selection and uninterrupted culture; CON, routine morphological embryo selection and interrupted culture.

Mean (SD) weight at birth was 3413 (588) g in the TLE group, 3412 (588) g in the TLR group, and 3377 (578) g in the CON group (*P* = 0.70). Regression analyses of birth weight percentiles of boys and girls born after fresh and FETs indicated no differences between the three treatment groups (Fig. 1). The proportions of children born with low and very low birth weight were comparable between the groups. Taken together, when the randomization group was not considered, children born after fresh embryo transfer had an average birth weight of 3360 (541) g, after FET 3476 (613) g, and after natural conception 3351 (543) g.

Mean (SD) gestational age at birth was 39.4 (1.9) weeks in the TLE group, 39.5 (1.5) weeks in the TLR group, and 39.3 (1.9) weeks in the CON group. Mean Apgar score after delivery was comparable between the groups.

Major congenital malformations occurred in four children in the TLE group, four in the TLR group, and seven in the CON group. Minor congenital malformations occurred in five children in the TLE group, three in the TLR group, and five in the CON group.

Health problems immediately after delivery were reported for eight babies in the TLE group, 12 in the TLR group, and 11 in the CON group.

Of all 13 twin pregnancies, five occurred after fresh embryo transfer (4 SET, 1 DET), six after FET (6 SET), and two were natural conceptions.

## Discussion

In this study, we examined the obstetric and perinatal outcomes of a multicentre RCT on TLM. We found that uninterrupted culture in a TLM incubator with and without the application of a machine learning embryo selection algorithm is safe in terms of obstetric and perinatal results compared to interrupted culture and morphological selection.

The incidence of serious pregnancy complications overall was very similar in the three groups. Pre-eclampsia was more often observed in the TLR group but the age-adjusted RR did not indicate a significant difference compared to the CON group. Two other studies have also found no indication that obstetric risks, including hypertensive disorders, are higher with TLM (Insua *et al.*, 2017; Ahlstrom *et al.*, 2023).

The proportion of women who had a spontaneous start of the delivery, induced delivery, ventouse delivery, or caesarean section was similar in the three groups. Previous studies only compared the number of caesarean sections between time-lapse and standard incubation. A retrospective analysis of data from an RCT found a lower number of caesarean sections in the time-lapse group (Insua *et al.*, 2017), while a large population-based retrospective analysis reported no differences for the number of caesarean sections (Ahlstrom *et al.*, 2023).

The proportion of preterm and very preterm births was comparable in the three groups, which is consistent with evidence from most earlier studies (Insua *et al.*, 2017; Kovacs *et al.*, 2019; Ma *et al.*, 2022; Ahlstrom *et al.*, 2023). One study found a lower risk for preterm births in the time-lapse group, but the retrospective cohort study design of this report should be considered when interpreting the results (Mascarenhas *et al.*, 2019).

No significant differences were observed between the three groups for average birth weight. The proportion of children born with a low and very low birth weight was also comparable across groups. Five other studies have also reported no differences between time-lapse and standard incubation regarding birth weight (Insua *et al.*, 2017; Kovacs *et al.*, 2019; Ma *et al.*, 2022; Ahlstrom *et al.*, 2023; Bhide *et al.*, 2024). The two studies, which reported a higher mean birth weight in the TLM group compared to conventional incubation should be interpreted with caution: the first because of its retrospective design (Mascarenhas *et al.*, 2019) and the second because of the use of double embryo transfers (Guo *et al.*, 2021). When data from all three treatment groups were pooled, our study confirmed the findings from earlier meta-analyses, which showed that children born after fresh embryo transfer have a lower average birth weight compared to children born after FET (Maheshwari *et al.*, 2018; Zaat *et al.*, 2021). It has been proposed that the lower birth weight in children born after fresh transfer may be related to ovarian stimulation during the oocyte retrieval cycle and corresponding hormonal changes that affect the uterine environment (van Duijn *et al.*, 2021). Gestational age at delivery was similar in the three groups, as was also described by six other studies (Insua *et al.*, 2017; Kovacs *et al.*, 2019; Mascarenhas *et al.*, 2019; Ma *et al.*, 2022; Ahlstrom *et al.*, 2023; Bhide *et al.*, 2024).

The health of children born in this study was not affected by randomization to the TLE, TLR, or CON group. Health problems directly after delivery, Apgar scores, the rates of major and minor congenital malformations, late miscarriages, and stillbirth/intrauterine deaths were all found to be comparable in all three groups. These findings are reassuring and generally in line with other studies (Insua *et al.*, 2017; Ma *et al.*, 2022; Ahlstrom *et al.*, 2023; Bhide *et al.*, 2024).

Strengths of our study include the broad inclusion criteria and multicentre design. Furthermore, our results are very robust, since each of the five laboratories used their own laboratory protocols, incubator settings, culture media, standard dishes, and cryopreservation techniques. Moreover, patients received a SET in almost all cases, with the exception of 18 of 1731 patients (1%) who changed their mind after randomization. Most pregnancies involved singleton births. Thirteen twin deliveries, 10 of which followed SET, were excluded from the analysis due to the increased risk of maternal and perinatal complications associated with multiple pregnancies (Rao *et al.*, 2004).

In our study, embryos in the TLE and TLR groups were cultured in micro-wells, which shared a common culture medium well, whereas embryos in the CON group were cultured in individual medium droplets. While such methodological differences in embryo culture conditions may influence embryo development, they do not appear to have affected our clinical outcomes. Findings from previous studies on group versus individual culture have not been consistent, with some reporting an effect on clinical outcomes and others observing no significant difference (Ebner *et al.*, 2010; Fancsovits *et al.*, 2022; Herreros *et al.*, 2024). In our study, embryos in all groups were cultured in the same time-lapse incubator. The control group reflected embryo culture conditions in a benchtop incubator. This approach had the advantage of a direct and clear comparison of uninterrupted and interrupted culture conditions and embryo selection methods, while minimizing interference from other incubator variables. However, a limitation of our control group is that our results cannot be used to draw conclusions about the safety of TLM compared to big box incubators. Embryos of multiple patients are cultured simultaneously in big box incubators, which leads to more door openings and thereby disturbances compared to benchtop incubators. However, the available data from three other RCTs also suggest that TLM is safe to use in comparison to several conventional incubators (Insua *et al.*, 2017; Kovacs *et al.*, 2019; Bhide *et al.*, 2024).

Another limitation of our study is that we only tested one time-lapse incubator, while more systems are currently available. It is advisable to report the safety of this add-on technique for each of the available time-lapse systems. Obstetric and perinatal data were recorded by research personnel based on hospital records. We also obtained data from patients with an online questionnaire. Discrepancies in data retrieved from questionnaires were carefully cross-checked with information recorded by research personnel to ensure consistency between both data sources, because self-reported data can be less accurate. In total, 72% of the patients who had a singleton ongoing pregnancy replied to our questionnaires. APGAR scores were retrieved from patient questionnaire only and therefore, this outcome should be considered with care. Power calculations were conducted for primary outcomes of the RCT. We are aware that imprecision for many of the comparisons reported here is sizeable.

In conclusion, our study shows that uninterrupted embryo culture with or without a TLM selection algorithm based on machine learning does not pose any obstetric or perinatal risks when compared to routine interrupted culture and morphological embryo selection. However, long-term data are not yet available and therefore, potential epigenetic effects, which may manifest later in life, cannot be excluded based on our data.

## Data Availability

Restricted access to the study data can be arranged on request to the corresponding author. Written proposals will be assessed by the SelecTIMO study group. A data sharing agreement including terms and conditions for authorship and publication will have to be signed before making the data available.
